# Infection kinetics, syncytia formation, and inflammatory biomarkers as predictive indicators for the pathogenicity of SARS-CoV-2 Variants of Concern in Calu-3 cells

**DOI:** 10.1371/journal.pone.0301330

**Published:** 2024-04-03

**Authors:** Priyo Budi Purwono, Vimvara Vacharathit, Suwimon Manopwisedjaroen, Natali Ludowyke, Ampa Suksatu, Arunee Thitithanyanont

**Affiliations:** 1 Faculty of Science, Department of Microbiology, Mahidol University, Bangkok, Thailand; 2 Faculty of Medicine, Department of Microbiology, Universitas Airlangga, Surabaya, Indonesia; 3 Faculty of Science, Systems Biology of Diseases Research Unit, Mahidol University, Bangkok, Thailand; 4 Faculty of Science, Department of Microbiology, Pornchai Matangkasombut Center for Microbial Genomics, Mahidol University, Bangkok, Thailand; Korea Disease Control and Prevention Agency, REPUBLIC OF KOREA

## Abstract

The ongoing COVID-19 pandemic has led to the emergence of new SARS-CoV-2 variants as a result of continued host-virus interaction and viral genome mutations. These variants have been associated with varying levels of transmissibility and disease severity. We investigated the phenotypic profiles of six SARS-CoV-2 variants (WT, D614G, Alpha, Beta, Delta, and Omicron) in Calu-3 cells, a human lung epithelial cell line. In our model demonstrated that all variants, except for Omicron, had higher efficiency in virus entry compared to the wild-type. The Delta variant had the greatest phenotypic advantage in terms of early infection kinetics and marked syncytia formation, which could facilitate cell-to-cell spreading, while the Omicron variant displayed slower replication and fewer syncytia formation. We also identified the Delta variant as the strongest inducer of inflammatory biomarkers, including pro-inflammatory cytokines/chemokines (IP-10/CXCL10, TNF-α, and IL-6), anti-inflammatory cytokine (IL-1RA), and growth factors (FGF-2 and VEGF-A), while these inflammatory mediators were not significantly elevated with Omicron infection. These findings are consistent with the observations that there was a generally more pronounced inflammatory response and angiogenesis activity within the lungs of COVID-19 patients as well as more severe symptoms and higher mortality rate during the Delta wave, as compared to less severe symptoms and lower mortality observed during the current Omicron wave in Thailand. Our findings suggest that early infectivity kinetics, enhanced syncytia formation, and specific inflammatory mediator production may serve as predictive indicators for the virulence potential of future SARS-CoV-2 variants.

## Introduction

The coronavirus disease 2019 (COVID-19) pandemic has produced a significant burden on global health and economies. Since its emergence, the causative agent severe acute respiratory syndrome coronavirus 2 (SARS-CoV-2) has spread to approximately 230 countries and territories, resulting in over 773 million confirmed cases and more than 6.9 million deaths by December 2023 [1]. SARS-CoV-2 is a highly contagious virus that primarily spreads through respiratory droplets. In addition to its high transmissibility, the virus has demonstrated an ability to evolve rapidly, resulting in the emergence of new variants of concern (VOCs) with distinct mutations scattered across the viral genome [2]. The World Health Organization (WHO) classifies VOCs based on their potential impact on global public health. When compared to the wild-type strain, these variants are generally associated with an increase in infectiousness, a greater virulence or disease presentation, or a decrease in the effectiveness of diagnostics, vaccines, and therapeutics [3]. The first notable mutation was a single spike substitution, the D614G mutation, that was first identified in January 2020 and is now present in all SARS-CoV-2 VOCs [4]. This early mutation type has been recognized to significantly generate a variant with more functional S protein and increase virus infectivity compared to wild-type [5]. Additionally, five VOCs have since been recognized, including Alpha, Beta, Gamma, Delta, and Omicron (Pango lineages B.1.1.7, B.1.351, P1, B.1.617.2, and B.1.1.529, respectively) [3,4]. Studies assessing the impact of these variants on the severity levels of COVID-19 have yielded inconsistent findings [6–10].

To better understand the pathogenicity related to SARS-CoV-2 VOC-specific variations, it is important to further delve into the cellular and molecular mechanisms of viral infection and spread. SARS-CoV-2 gains entry into host cells through the interaction between its spike (S) protein to angiotensin-converting enzyme 2 (ACE2) receptors present in various human cell types [11–13]. Upon binding, TMPRSS2 –a type II transmembrane serine protease also expressed on certain host cells–enhances the proteolytic cleavage between the S1/S2 subunits and S2’ site, promoting viral entry via plasma membrane fusion [14]. The TMPRSS2-dependent plasma membrane pathway is thought to be the most crucial entry pathway used by SARS-CoV-2 variants [15], although the virus is also capable of entering host cells via the cathepsin-L-dependent endosomal pathway [16,17]. Additionally, lung epithelial cells express both ACE2 and TMPRSS2 and are recognized as the primary site of SARS-CoV-2 entry [11,18]. These cells are among the first to initiate a host cellular response during infection and can act as a reservoir for sustained viral replication, effectively contributing to viral persistence and pathology [19]. In several studies demonstrated the Delta variant infects the Vero-E6 with TMPRSS2 overexpressed cells at a higher rate compared to wild-type and Omicron variant [20,21]. However, the infectivity and growth kinetic of each variant in human lung epithelial cells are required to evaluate.

SARS-CoV-2 infection can lead to severe consequences in the lungs, such as Acute Respiratory Disease Syndrome (ARDS), which exhibits distinct pathological characteristics [22], including the morphological change in host cells resulting from virus infection named as cytopathic effect (CPE) [23]. According to the histopathological data from autopsy, syncytia cells are the predominant CPE found in the lung tissue damage in 20 out of 41 COVID-19 patients and strongly related to severe pneumonia cases [24]. The formation of syncytia is triggered by cell-to-cell fusion which allows the merge of the cell membrane and cytoplasmic materials of two or more cells generating multinucleated giant cells [25]. Initial studies have revealed that the S protein is expressed on the surface of SARS-CoV-2 infected cells, with the majority of S protein synthesis, processing, and viral budding occurring within intracellular membranes [26,27]. Additionally, the interaction between the ACE2 receptors on neighboring cells and the S proteins on the surface of the infected cell leads to the formation of syncytia [28]. This process is further enhanced by the presence of the TMPRSS2 protease to activate S protein by cleavage at furin cleavage site of S protein into two subunits, S1 and S2 subunits [29]. Furthermore, a previous study has revealed that Vero-E6 cells infected with SARS-CoV-2 spike–expressed pseudotype virus exhibited more and larger syncytia cells compared to those expressing the SARS-CoV spike [30]. These results indicate a notable difference in the formation of syncytia might also be induced by different variants with distinct genomic mutations gene.

As an additional pathological consequence, infection with SARS-CoV-2 can also trigger a cascade of events leading to the secretion of inflammatory immune mediators which, if dysregulated, may result in lung damage [31,32]. The immune response to SARS-CoV-2 is initiated through the detection of viral single-stranded RNA (ssRNA) by pattern recognition receptors (PRRs), such as RIG-1-like receptors (RLRs) and Toll-like receptors (TLRs), leading to cytokine production, including antiviral interferons (IFNs) and other pro- and anti-inflammatory cytokines [33]. An excessive accumulation of immune cell infiltrates and cytokines can lead to ARDS or extrapulmonary multiple-organ failure in severe cases [33,34]. Furthermore, several studies have uncovered a correlation between the patterns of cytokines and chemokines in COVID-19 patients and disease severity, particularly COVID-19 patients with severe pneumonia exhibit higher distinct cytokines, including IP-10, IL-6, IL-7, and VEGF, in comparison to those individuals presenting mild and moderate symptoms [35,36]. Another study has expanded results with elevated levels of plasma cytokines (IL-2, IL-7, IL-10, G-CSF, IP-10, MCP-1, MIP-1α, and TNF-α) in COVID-19 patients requiring Intensive Care Unit (ICU) admission compared to non-ICU patients [31].

The Calu-3 cell line, expressing both ACE2 and TMPRSS2 [37–39], is highly permissive to SARS-CoV-2 infection and can be served as a critical tool for the study various aspects of SARS-CoV-2 VOCs, including virus infectivity, pathogenesis, evolution, and targeted drug development [37,40–43]. Despite numerous studies on the characterization of SARS-CoV-2 infection with different VOCs, there is no direct comparison among all variants covering virological, cellular impact and innate immune response profiles. Consequently, our study aimed to analyze essential pathogenicity parameters in human lung epithelial cells, including infectivity rate, replication kinetics, syncytia formation, cytopathic effects, and innate immune response, encompassing six distinct SARS-CoV-2 variants. We infected Calu-3 with SARS-CoV-2 WT, D614G, Alpha, Beta, Delta, and Omicron variants and compared viral kinetics, syncytia formation, and cytokine induction. Our results showed that D614G, Alpha, Beta, and Delta variants exhibited more efficient virus entry than the wild-type virus with similar replication kinetics and peak virion production occurring within 48 hours post-infection. The Delta variant induced the highest level of syncytia formation, followed by the Alpha variant. Conversely, the Omicron variant exhibited a lower infectivity rate, slower replication kinetics, and fewer syncytia formations compared to other variants. Additionally, the Delta variant induced significantly higher expression of specific inflammatory cytokines and growth factors, indicating its potential for high pathogenicity in lung epithelial cells, as opposed to the Omicron variant which showed weaker induction of these inflammatory mediators. Our results suggest that comparative *in vitro* studies of SARS-CoV-2 variant infection kinetics, syncytia formation, and inflammatory biomarkers may serve as useful predictive indicators for variant pathogenicity in future outbreak preparedness.

## Materials and methods

### Cell culture

Four cell lines were cultured in this study. (i) Vero cells (ATCC^®^ CCL-81^™^) African green monkey (*Cercopithecus aethiops*) kidney epithelial cells, were cultured in Modified Eagle Medium (MEM) high glucose (Gibco, USA) with 10% fetal bovine serum (FBS) (Gibco, USA), 1x MEM Non-Essential Amino Acid (Gibco, USA) and 100 μg/mL penicillin/streptomycin (Invitrogen, USA). **(ii)** Vero E6 cells (ATCC^®^CRL-1586^™^) were maintained in Dulbecco’s Modified Eagle Medium (DMEM) high glucose (Gibco, USA) with 10% FBS (Gibco, USA) and 100 μg/mL penicillin/streptomycin (Invitrogen, USA). **(iii)** Vero E6 cells with a stably high expression of TMPRSS2 (Vero E6/TMPRSS2 cells) were obtained from JCRB Cell Bank in Japan (JCRB number JCRB1819). The cells were cultured in DMEM low glucose (Gibco, USA), supplemented with 10% FBS (Gibco, USA), G418 (1 mg/mL) (Nacalai Tesque, Japan), and 100 μg/mL penicillin/streptomycin (Invitrogen, USA). **(iv)** Human airway epithelial cells, Calu-3, were obtained from American Type Culture Collection (ATCC^®^HTB-55^™^). The cells were cultured in Dulbecco’s Modified Eagle Medium: Nutrient Mixture F-12 (DMEM/F-12) (Gibco, USA) with 10% FBS (Gibco, USA), 100 μg/mL penicillin/streptomycin (Invitrogen, USA) and 1% GlutaMAX (Gibco, USA). All cultures were maintained in a humidified incubator at 37 °C and with 5% CO_2_.

### Virus isolation, propagation, and titration

All the SARS-CoV-2 virus isolates were collected from nasopharyngeal swabs in COVID-19 patients in Thailand. The earlier variant, wild-type (WT), was isolated in Vero cells, then propagated twice in Vero E6 cells for working stock. Furthermore, the later variants, B.1.36.16/D614G, B.1.1.7/Alpha, B.1.351/Beta, B.1.617.2/Delta, and B.1.1.529.2.10/Omicron (BA.2.10), were isolated in Vero E6/TMPRSS2 cells in the presence of 2% FBS (Gibco, USA) and 100 μg/mL penicillin/streptomycin (Invitrogen, USA). The cytopathic effects were observed under an inverted microscope. The presence of SARS-CoV-2 isolates was further confirmed by the detection of the viral genome in the culture supernatant by real-time RT-PCR. To prepare the working stock of these variants, two further virus passages were performed in Vero E6/TMPRSS2 cells in order to minimize virus propagation-related mutations [39,41]. Briefly, 100 μl of the isolated virus is added into Vero E6/TMPRSS2 (1.0x10^7^ cells in T-75 flask), then incubate at 37°C for one hour. After that, the culture medium is replaced with DMEM low glucose, containing 2% FBS (Gibco, USA) and 100 μg/mL of penicillin/streptomycin (Invitrogen, USA). When the observed cytopathic effect (CPE) was substantial, approximately at 75% or more (corresponds to a grading of 3+ or higher compared to uninfected cells) which characterized by cell morphological changes such as rounding, deteachment, lysis and syncytia formation, culture supernatant was collected and centrifuged at 3000 rpm at 4°C for 10 minutes to separate cell debris. The supernatant was then aliquoted and kept as a working virus stock.

To quantitate the titer of prepared working stock, a plaque assay was performed. In brief, 24 hours before infection, the Vero E6/TMPRSS2 cells monolayer was seeded in a 6-well plate. A serial dilution of virus stock was inoculated into the cells and incubated for viral adsorption at 37°C for one hour. Then, an overlay medium containing 2% FBS and 1% agarose in DMEM Low Glucose media was added into the well. The culture was incubated at 37°C in 5% CO_2_ for three days. Thereafter, plaque phenotypes were visualized by staining with 0.33% Neutral Red solution (Sigma-Aldrich, USA) for 5 hrs. Plaque numbers were counted and the virus titers were calculated as plaque-forming units per milliliter (PFUs/mL). All the SARS-CoV-2 isolation, propagation, and experiments were performed at a certified Biosafety Level 3 facility at the Faculty of Science, Mahidol University.

### Identification SARS-CoV-2 VOCs

SARS-CoV-2 VOCs of all virus isolates were determined by using whole genome sequencing. The whole genome sequencing protocol was following the previous study [44]. The consensus sequences of the spike protein of the wild-type (WT), B.1.1.7 (Alpha), B.1.351 (Beta), and B.1.617.2 (Delta) have been deposited in GenBank and can be obtained under the accession numbers: SARS-CoV-2/human/THA/LJ07_P3/2020 MZ815437, SARS-CoV-2/human/THA/NH088_P3/2021 MZ815440, SARS-CoV-2/human/THA/OTV007_P3/2021 MZ815439, and SARS-CoV-2/human/THA/NH657_P3/2021 MZ815438, respectively. In addition, B.1.36.16/D614G and B.1.1.529.2.10 (Omicron/BA.2.10) genome sequences were submitted on GSAID with accession number: EPI_ISL_15125439 and EPI_ISL_16355925, respectively.

### SARS-CoV-2 infection

Calu-3 cells were seeded at 3 × 10^4^ cells/well in a 96-well plate (Corning, USA) and left to adhere at 37 °C, 5% CO_2_ for two days to reach approximately 70% of cell confluence. Cells were washed with 1x phosphate-buffered saline (PBS) and adsorbed with each variant of SARS-CoV-2 at the multiplicity of infection (MOI) of 0.1 at 37 °C for two hours, additionally, Calu-3 media only was also used for mock-infected condition. The viral inoculum was then removed, and cells were washed twice with 1x PBS. A fresh culture (DMEM/F12 with 10% FBS, 1x GlutaMAX, and 100 μg/mL penicillin/streptomycin) was added and the culture was maintained at 37°C in the presence of 5% CO_2_. At 12, 24, 48, and 72 hours post-infection, culture supernatants, as well as the infected cells were harvested and fixed for evaluation of the viral output by foci forming assay and for detection of the viral nucleocapsid protein (NP) expression by immunofluorescence staining, respectively. All experiments involving virus infection were performed in triplicate.

### Immunofluorescence staining of SARS-CoV Nucleocapsid protein (NP)

Infected cells were fixed with 4% paraformaldehyde for one hour at room temperature, then permeabilized by 0.5% triton-x in 1xPBS for 15 minutes at room temperature. After that, the plate was blocked by 2% (w/v) Bovine Serum Albumin (BSA) in PBST (0.05% Tween-20) for one hour at room temperature. For the primary staining of viral protein, rabbit anti-SARS-CoV NP monoclonal antibody (Sino Biological Inc., China) at a 1:500 ratio was added into the cells and incubated at 37 °C for one hour. The cells were washed with phosphate-buffered saline containing 0.05% Tween (PBST) three times and incubated with secondary antibodies with a 1:500 ratio of goat anti-rabbit Alexa Fluor^™^ 488 (Invitrogen, USA). After washing, cellular nuclei were stained with Hoechst dye (Thermo Fisher Scientific, USA). BioTek Cytation 7 Cell Imaging Multi-Mode Reader (Agilent Technologies, USA) was used to detect fluorescent signals. The percentage of infected cells and the number of syncytial cells, based on fluorescently labeled NP protein, were randomly measured from eight images at 10x magnification per well and analyzed by Gen5 Microplate Reader and Imager software version 3.11 (Agilent Technologies, USA). Furthermore, the determination of syncytia type was based on the number of observed nuclei in the formation of fused infected cells, with categorization into small syncytia (5–10 nuclei) and large syncytia (more than 10 nuclei).

### Focus-forming assay

This assay was performed to determine the number of infectious particles or viral output released from the infected cells. In brief, 24 hours before infection, Vero E6/TMPRSS2 cells were seeded in a 96-well at 2x10^4^ cells/well. The cells were infected with a serial dilution of the virus-containing supernatants for one hour at 37°C. Then, the cells were overlaid with 100 μL/well of overlay medium containing DMEM low glucose (Gibco, USA) supplemented with 2% FBS (Gibco, USA) and 2% carboxymethylcellulose sodium salt (CMC) (Sigma-Aldrich, USA). The culture was incubated at 37°C in 5% CO_2_ for 20 hours. The cell monolayer was fixed with 4% formaldehyde for one hour and then permeabilized in 0.5% Triton X-100 at room temperature for 15 minutes. Cells were then incubated with 1:2500 rabbit monoclonal antibody against SARS-CoV-2 nucleocapsid protein (Sino Biological, China) at room temperature for one hour, followed by incubation with 1:1000 horseradish peroxidase-conjugated goat anti-rabbit IgG secondary antibody (Agilent Technologies, USA) for 1h. The cells were stained with True-Blue peroxidase substrate (SeraCare Life Science, USA) for 10 min in a dark chamber. Foci numbers were observed under BioTek Cytation 7 Cell Imaging Multimode Reader (Agilent Technologies, USA) and analyzed by Gen5 Microplate Reader and Imager software version 3.11 (Agilent Technologies, USA). The foci were counted and calculated as focus-forming units per milliliter (FFUs/mL).

### Measurement of cytokines/chemokines

Viruses in supernatant samples are inactivated with 0.5% Triton X-100 for 1 hour at room temperature. The levels of immune mediators, including cytokines, chemokines, and growth factors were quantified using the Milliplex Human Cytokine/Chemokine/Growth Factor Panel A (Merck Millipore, USA) following the manufacturer’s instructions. This system allowed quantitative measurements for 25 biomarkers: FGF-2, G-CSF, GM-CSF, IFN-α2, IFN- γ, IL-1 β, IL-1RA, IL- 2, IL-3, IL-6, IL-7, IL-8, IL-9, IL-10, IL-12 p70, IL-17A, IL-22, IP-10, MCP-1, MIP-1α, MIP-1β, PDGF-AA, PDGF-BB, TNF-α, and VEGF-A. The analysis was performed on a Luminex MAGPIX (Luminex Multiplexing Instrument, Merck Millipore) with a minimum of 50 beads collected per analyte per well. Belysa software was used to generate standard curves for each analyte using five parametric logistic fit models. Statistical data analysis was performed by using the one-way ANOVA Kruskal Wallis test with Dunn’s multiple comparison test and figures were generated using GraphPad Prism 8.0 (GraphPad Software Inc, USA).

### Statistical analyses

Statistical analyses of data and the creation of graphs were performed with GraphPad Prism version 8 (GraphPad Software, San Diego, CA, USA). To investigate the significance of differences of experimental virus isolates to the wild-type, a one-way ANOVA analysis followed by Dunnett’s multiple comparisons test was conducted. The standard deviation is depicted by the error bars (SD).

## Results

### Growth kinetics of SARS-CoV-2 variants isolated in Thailand

The six SARS-CoV-2 variants (WT, D614G, Alpha, Beta, Delta, and Omicron) analyzed in this study were selected based on their high prevalence during each successive COVID-19 wave in Thailand. According to genomic monitoring studies [45], the first wave (March—June 2020) was dominated by the wild-type (WT), then the second wave (December 2020—February 2021) was dominated by B.1.36.16, a non-VOC variant containing the D614G mutation (hereafter referred to as “D614G”). The third wave was dominated by Alpha (April–June 2021), while Beta was also detected in this third wave. Delta was the major variant during the fourth wave (July–December 2021), and Omicron during the fifth wave (January 2022—present). We first evaluated the growth kinetics of these six SARS-CoV-2 variants in human lung epithelial cells, specifically Calu-3 cells.

**Fig 1:**
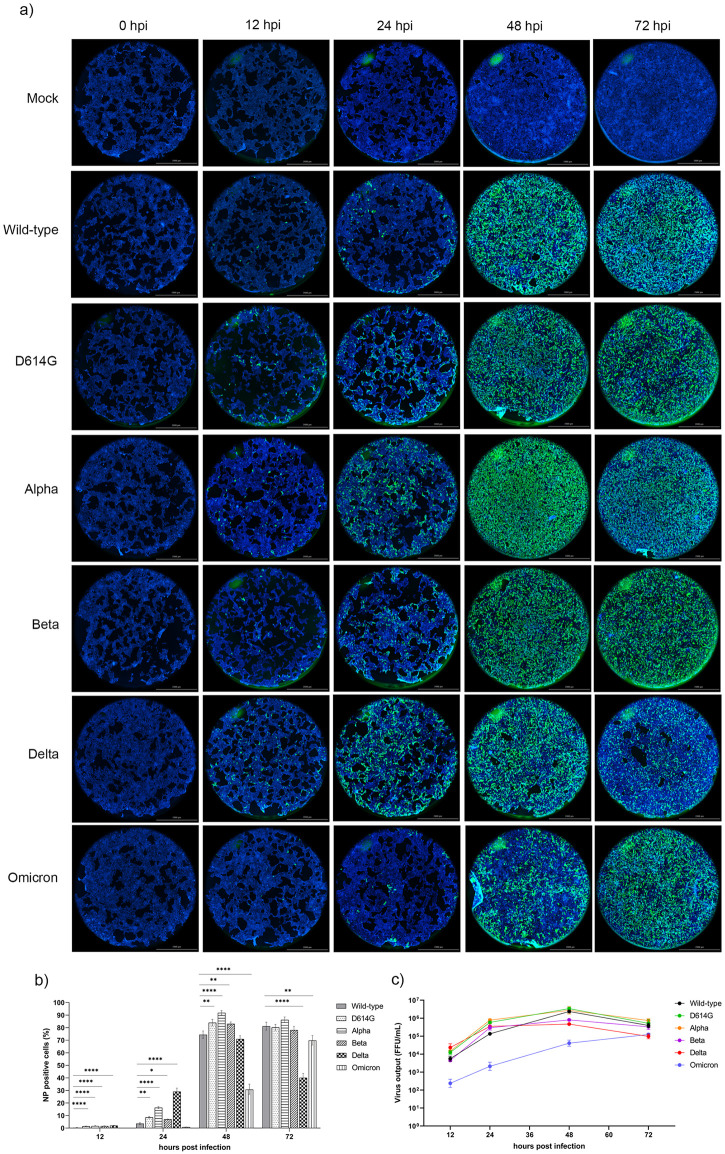
The growth kinetics of SARS-CoV-2 VOCs in Calu-3 cells. The cells were infected with each SARS-CoV-2 variant at MOI 0.1 for 2 hours and subsequently washed and incubated further for 12, 24, 48, and 72 hpi. At the indicated time point, the infected cells were then fixed and stained for viral nucleocapsid proteins with anti-SARS-CoV NP mAb. **(a)** Representative fluorescent images of the infected Calu-3 cells. The SARS-CoV-2 infected cells were detected by a 2.5x lens of BioTek Cytation 7 Cell Imaging Multi-Mode Reader (Agilent Technologies, USA) (SARS-CoV NP- Alexa Fluor^™^ 488: Green; Hoechst: Blue). Scale bar: 2000 μm. **(b)** The levels of the infected cells were calculated based on the expression of viral NP at different time points. Statistical analysis was performed by using one-way ANOVA with subsequent Dunnett’s multiple comparisons test. *p<0.05, **p<0.01, ***p<0.001, and ****p<0.0001. **(c)** The production of infectious virions at various time points was analyzed from the harvested culture supernatants at the indicated time points by foci-forming assay. All experiments were performed in triplicate.

Viral growth kinetics were initially evaluated based on immunofluorescent staining of viral nucleocapsid protein (NP) (Fig 1a). We noted that NP expression of all variants in Calu-3 cells began as early as 12 hours after infection. At this initial stage, the wild-type started with a modest 0.30% of NP-positive cells, however, all VOCs except Omicron demonstrated elevated levels of viral NP expression compared to the wild-type, indicating a more effective virus entry. Significantly, the Delta variant displayed the greatest proportion of NP-positive cells, followed by Alpha, Beta, and D614G variant at 2.02%, 1.70%, 1.69% and 1.49%, respectively. By 24 hours post infection, the Delta variant showed markedly rapid expression of viral protein, with approximately 29.18% NP-positive cells, followed by Alpha and D614G variant with 16.34% and 8.56% respectively. At the 48-hour time point, the Delta, Beta, D614G and Alpha variant reached their highest infectivity, with NP-positive cells at around 71.03%, 83.09%, 84.04% and 91.85%, respectively. However, by 72 hours, there was a noticeable decline in the infectivity levels of these variants. Conversely, the wild-type infection level increased dramatically to 74.50% at 48 hours, then slightly continued to 81.20% at 72 hours. Omicron variant also exhibited a much slower and gradual increase in NP expression with 30.97%, and 69.93% NP-positive cells at 48, and 72 hours, respectively, as depicted in Fig 1b.

Next, the focus-forming assay was used to evaluate the kinetics of infectious virion production in Calu-3 cells infected with different VOCs at different time points post-infection. Consistent with the immunofluoresecence assay results, viral titers of all SARS-CoV-2 variants except for Omicron peaked at 48 hpi and declined at 72 hours. Interestingly, despite a slower replication rate compared to the other variants, Omicron viral titers continued to increased up until the 72-hour endpoint (Fig 1c).

### Syncytia formation in Calu-3 cells

**Fig 2:**
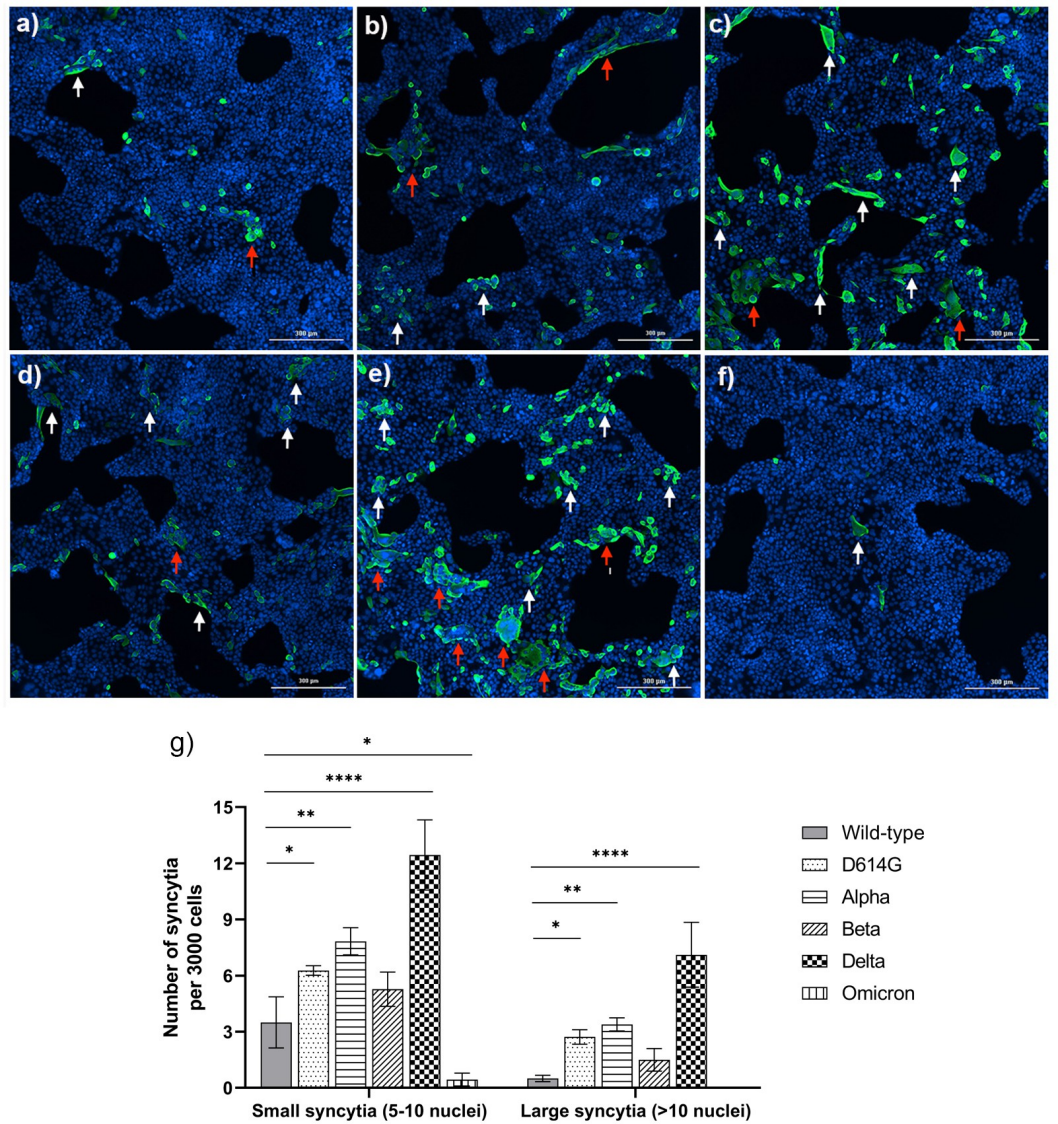
Representative fluorescent images of syncytia formation in Calu-3 cells induced by infection with different SARS-CoV-2 variants. The Calu-3 cells were infected with SARS-CoV-2 at MOI 0.1 including the wild-type strain **(a)**, D614G/B.1.36.16 **(b)**, Alpha/B.1.1.7 **(c)**, Beta/B.1.351 **(d)**, Delta/B.1.617.2 **(e)**, and Omicron/ B.1.1.529.2.10 (BA.2.10) **(f)**. Syncytia formation was monitored based on the expression of anti-SARS-CoV NP mAb at 24 hours post-infection. The images were observed under a 10x lens of BioTek Cytation 7 Cell Imaging Multimode Reader (Agilent Technologies, USA). (SARS-CoV NP- Alexa Fluor^™^ 488: Green; Hoechst: Blue). Scale bar: 300 μm. (g) The average number of syncytia per 3000 cells in different variants was demonstrated in a bar chart. Syncytia were grouped into two categories, small (5–10 nuclei, white arrow) and large syncytia (>10 nuclei, red arrow). The experiment was performed in triplicate. The statistics were analyzed by using one-way ANOVA with Dunnett’s multiple comparisons test. *p<0.05, **p<0.01, ***p<0.001, and ****p<0.0001.

**Fig 3:**
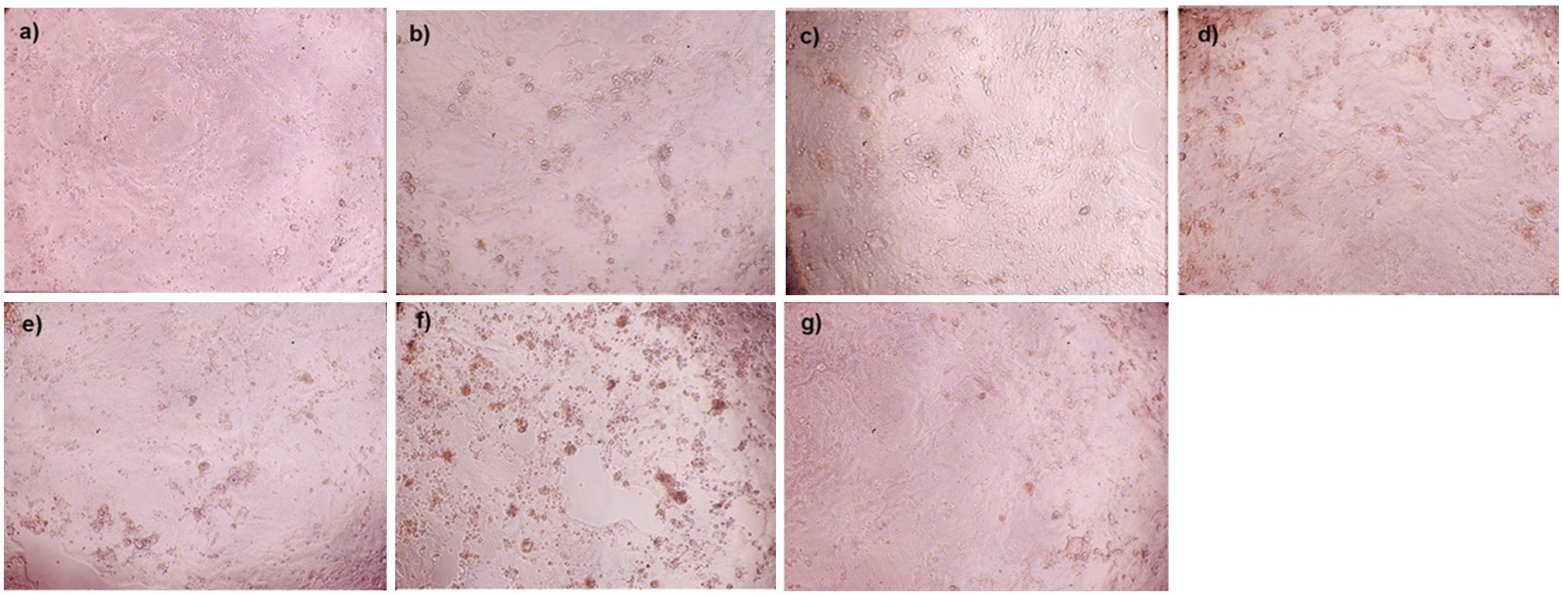
Cytopathic effect (CPE) upon SARS-CoV-2 VOCs infection in Calu-3 cells. The Calu-3 cells were infected with SARS-CoV-2 at MOI 0.1 for 48 hours. **(a)** Representative images of CPEs Calu-3 cells following infection with different SARS-CoV-2 variants; mock infection **(a)**, wild-type strain **(b)**, D614G/B.1.36.16 **(c)**, Alpha/B.1.1.7 **(d)**, Beta /B.1.351 **(e)**, Delta/B.1.617.2 **(f)**, and Omicron/ B.1.1.529.2.10 (BA.2.10) **(g)**. All images were taken at 100× magnification using the Eclipse TS100 inverted microscope (Nikon, USA). All experiments were performed in triplicate.

Formation of syncytia, triggered by the fusion of cell membranes and cytoplasmic materials of two or more cells, is recognized as one of the frequent CPE hallmarks in SARS-CoV-2 infected lungs [24]. We quantified syncytia formation in variant-infected Calu-3 cells at 24 hpi, which was the earliest and most appropriate time to observe this characteristic form (Fig 2). In comparison to cells infected with other variants, Calu-3 cells infected with the Delta variant exhibited notably elevated numbers of syncytia, including both small syncytia (5–10 nuclei) and large syncytia (more than 10 nuclei). Specifically, approximately 12.4 (±1.8) small syncytia and 7.1 (±1.7) large syncytia were observed per 3,000 Delta-infected cells. This was followed by infection with the Alpha variant, which resulted in 7.8 (±0.7) and 3.4 (±0.3) per 3,000 cells for small and large syncytia, respectively. However, the Omicron variant showed significantly fewer syncytia compared to the wild-type, with 0.4 (±0.3) small syncytia per 3,000 cells and no large syncytia observed. Meanwhile, the D614G variant induced significantly higher numbers of large syncytia, but comparable numbers of small syncytia in comparison to wild-type. Also, the size of syncytia formation due to infection with the Beta was comparable to that obtained from the wild-type. Compared to the other variants, the Delta variant caused more pronounced CPE at 48 hpi, as observed from the numerous rounded cells that have detached from the plate surface, known as floating syncytia [46,47]. In contrast, this was much less frequently observed in Omicron-infected Calu-3 cells (Fig 3).

### Profiles of inflammatory mediator production in Calu-3 cells

We next assessed the production of various immune mediators in Calu-3 cells infected with WT, D614G, Alpha, Beta, Delta, and Omicron variants at 48 hpi, a time point at which most variants displayed peak growth kinetics. The levels of the secreted cytokines, chemokines, and growth factors were measured in cell supernatants using a multiplex immunoassay panel which was selected in our previous study with COVID-19 patient’s clinical serum [35]. Among a total of 25 biomarkers, 23 were detectable.

**Fig 4:**
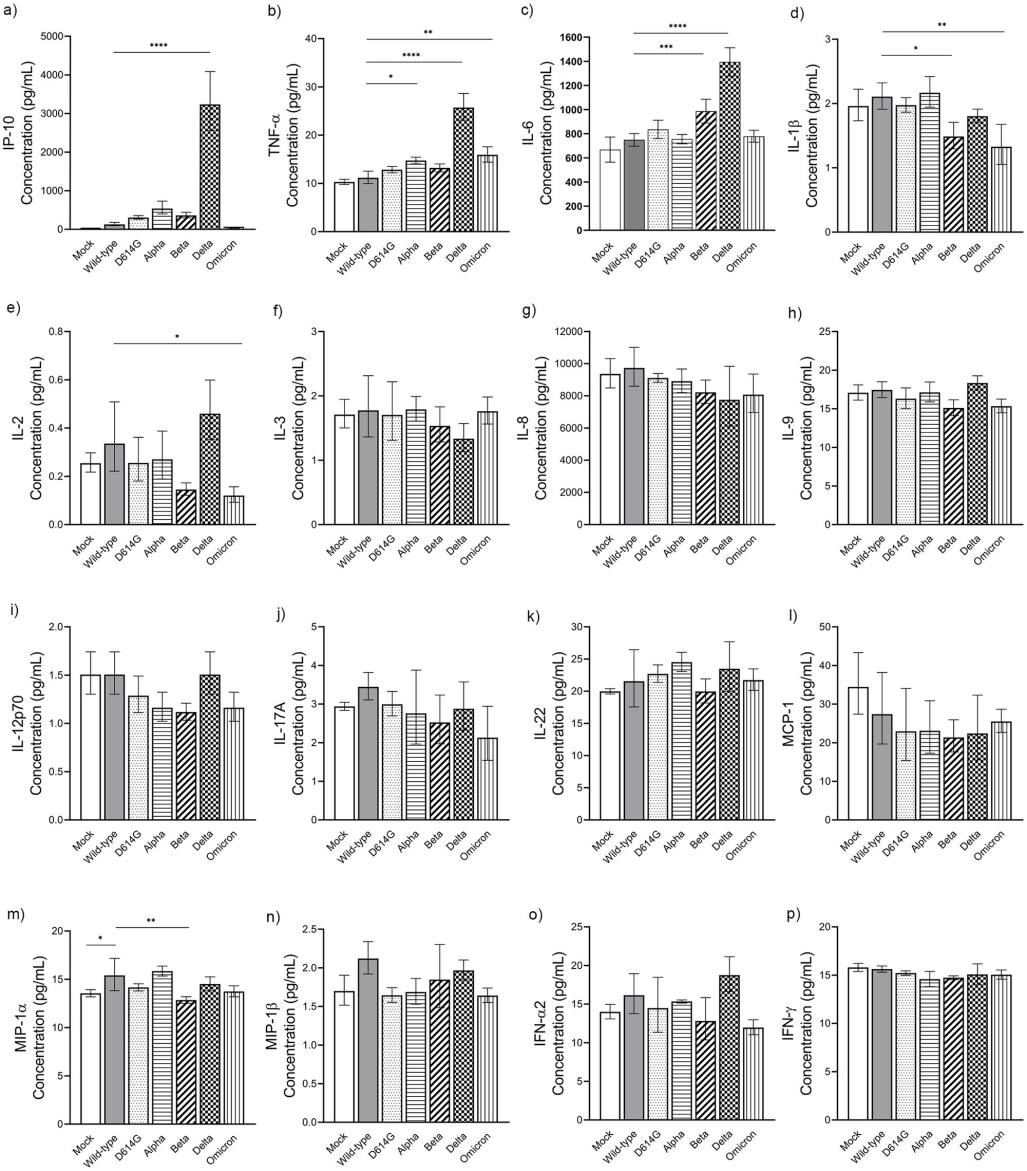
The measurement of pro-inflammatory cytokines/chemokines and interferons following infection Calu-3 cells with SARS-CoV-2 variants. The Calu-3 cells were infected with different SARS-CoV-2 variants at MOI 0.1. At 48 hpi, supernatant of each well were collected and measured for pro-inflammatory cytokine/chemokine concentration. The values of IP-10 **(a)**, TNF-α **(b)**, IL-6 **(c)**, IL-1β **(d)**, IL-2 **(e)**, IL-3 **(f)**, IL-8 **(g)**, IL-9 **(h)**, IL-12p70 **(i)**, IL-17A **(j)**, IL-22 **(k)**, MCP1 **(l)**, MIP-α **(m)**, MIP-β **(n)**, IFN-α2 **(o)**, and IFN-γ **(p)** were compared among variants. The geometric mean value of triplicate is shown with error bars representing the SD of the mean. Mock infection, wild-type, D614G, Alpha, Beta, Delta, and Omicron variants were presented in different bar motifs. Statistical significance was calculated using the one-way ANOVA with Dunnett’s multiple comparisons test. *p<0.05, **p<0.01, ***p<0.001, and ****p<0.0001.

Among the SARS-CoV-2 VOCs used in the Calu-3 cells model, there are different induction levels of each inflammatory cytokine type. Infection with the Beta variant had little impact on the production of Interferon γ-induced protein 10/IP-10 (C-X-C motif chemokine ligand 10/CXCL-10) and tumor necrosis factor-α (TNF-α) (Fig 4a and 4b), and resulted in a significant increase in Interleukin 6 (IL-6) levels (Fig 4c). However, this variant infection induced a lower amount of Interleukin 1β (IL-1β) and macrophage inflammatory protein-1α (MIP-1α) compared to the wild-type (Fig 4d and 4m), In the other hand, the Alpha variant triggered a considerable level of TNF-α (14.73 pg/mL) and a modest rise of IP-10 level than wild type (Fig 4a and 4b). Notably, the Delta variant was found to be the most potent inducer of several inflammatory mediators. IP-10 (CXCL10), TNF-α, and IL-6 were significantly upregulated in response to Delta infection, with concentrations of 3,236, 25.73, and 1,391 pg/mL, respectively (Fig 4a–4c, S1 Table). Furthermore, upon infection with this variant, a modest increase in interferon alpha2/IFN-α2 (18.74 pg/mL) was observed, but not with the other VOCs. In contrast, infection with the Omicron, yielded a slight increase in TNF-α secretion with less amount of IL-1β and Interleukin 2 (IL-2) compared to wild-type (Fig 4b, 4d and 4e, S1 Table).

**Fig 5:**
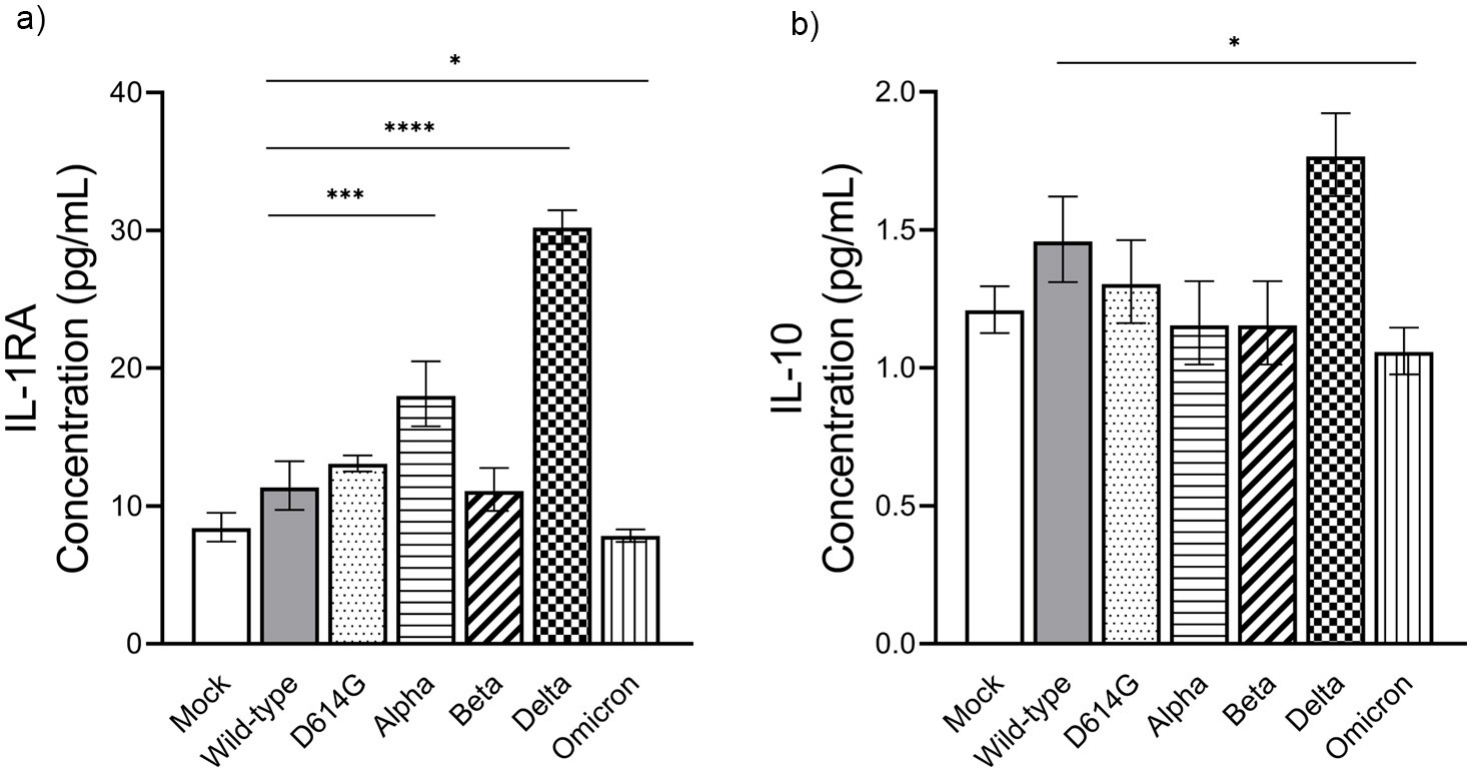
The measurement of anti-inflammatory cytokines following infection Calu-3 cells with SARS-CoV-2 variants. Different SARS-CoV-2 variants were used to infect the Calu-3 cells at a MOI of 0.1. Supernatants from each well were collected at 48 hpi, and their anti-inflammatory cytokine concentrations were analyzed. The values of IL-1RA **(a)** and IL-10 **(b)** were compared among VOCs. The geometric mean value of triplicates is shown with error bars representing the SD of the mean. Mock infection, wild-type, D614G, Alpha, Beta, Delta, and Omicron variants were presented in different bar motifs. Statistical significance was calculated using the one-way ANOVA with Dunnett’s multiple comparisons test. *p<0.05, ***p<0.001, and ****p<0.0001.

In the case of anti-inflammatory cytokines, no significant changes in biomarker levels were observed in Calu-3 cells infected with the SARS-CoV-2 D614G, Alpha, and Beta variants compared to the wild-type. However, Omicron variant infection yielded a considerable lower induction of anti-inflammatory markers than the wild-type control. Meanwhile, the Delta variant induced significantly elevated levels of Interleukin-1 receptor antagonist (IL-1RA) (30.21 pg/mL) compared to the wild-type, followed by the Alpha variant with concentration 17.99 pg/mL. In addition, levels of interleukin-10 (IL-10) were slightly greater (1.77 pg/mL) in Delta-infected cells than in cells infected with other variants (Fig 5, S1 Table), although this was not statistically significant. These results underscore the Delta variant’s capacity to induce the production of both pro- and anti-inflammatory mediators upon infecting human lung epithelial cells, highlighting its immunomodulatory potential.

**Fig 6:**
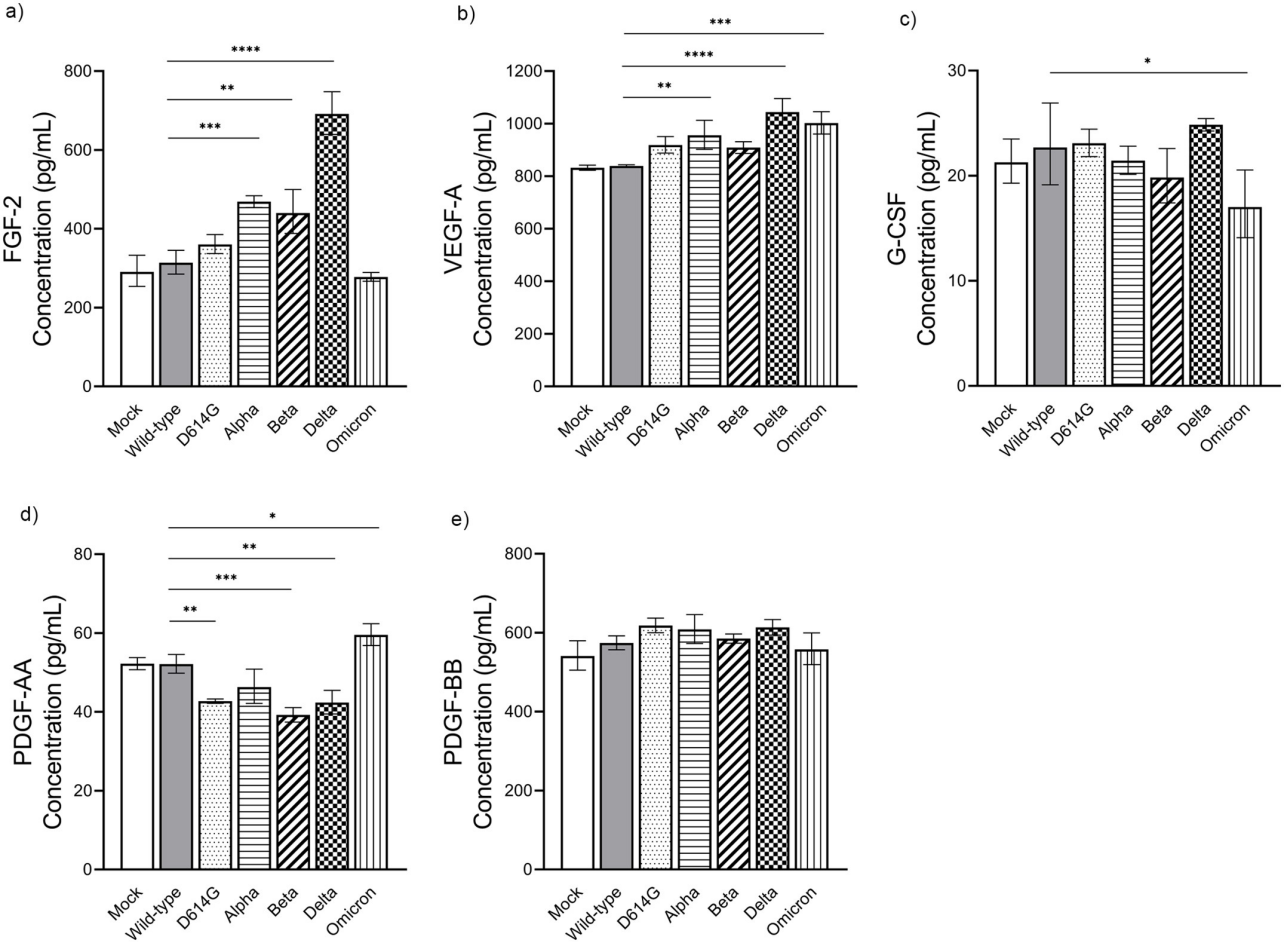
The measurement of secreted growth factors following infection Calu-3 cells with SARS-CoV-2 variants. The Calu-3 cells were infected with various SARS-CoV-2 variants at MOI 0.1. The supernatant of each well were taken at 48 hpi and measured for growth factors concentration. The values of FGF-2 **(a)**, VEGF-A **(b)**, G-CSF (c), PDGF-AA **(d)**, and PDGF-BB **(e)** were compared among variants. The geometric mean value of triplicates is shown with error bars representing the SD of the mean. Mock infection, wild-type, D614G, Alpha, Beta, Delta, and Omicron variants were presented in different bar motifs. Statistical significance was calculated using the one-way ANOVA with Dunnett’s multiple comparisons test. *p<0.05, **p<0.01, ***p<0.001, and ****p<0.0001.

Among the five growth factors included in the assay, infection with the Omicron variant moderately upregulated the expression of vascular endothelial growth factor-A (VEGF-A) and platelet-derived growth factor-AA (PDGF-AA). However, there was a lower induction of granulocyte colony-stimulating factor (G-CSF) by this variant compared to the wild-type. In the other hand, the Alpha variant infection yielded an elevated level of VEGF-A (955.80 pg/mL). Meanwhile, infection with the Delta variant resulted in significant upregulation of the fibroblast growth factor 2 (FGF-2) and VEGF-A, with concentrations of 691.40 and 1,044.00 pg/mL, respectively. Further, we observed a small increase in G-CSF concentration post-Delta infection (24.85 pg/mL) (Fig 6, S1 Table).

**Fig 7:**
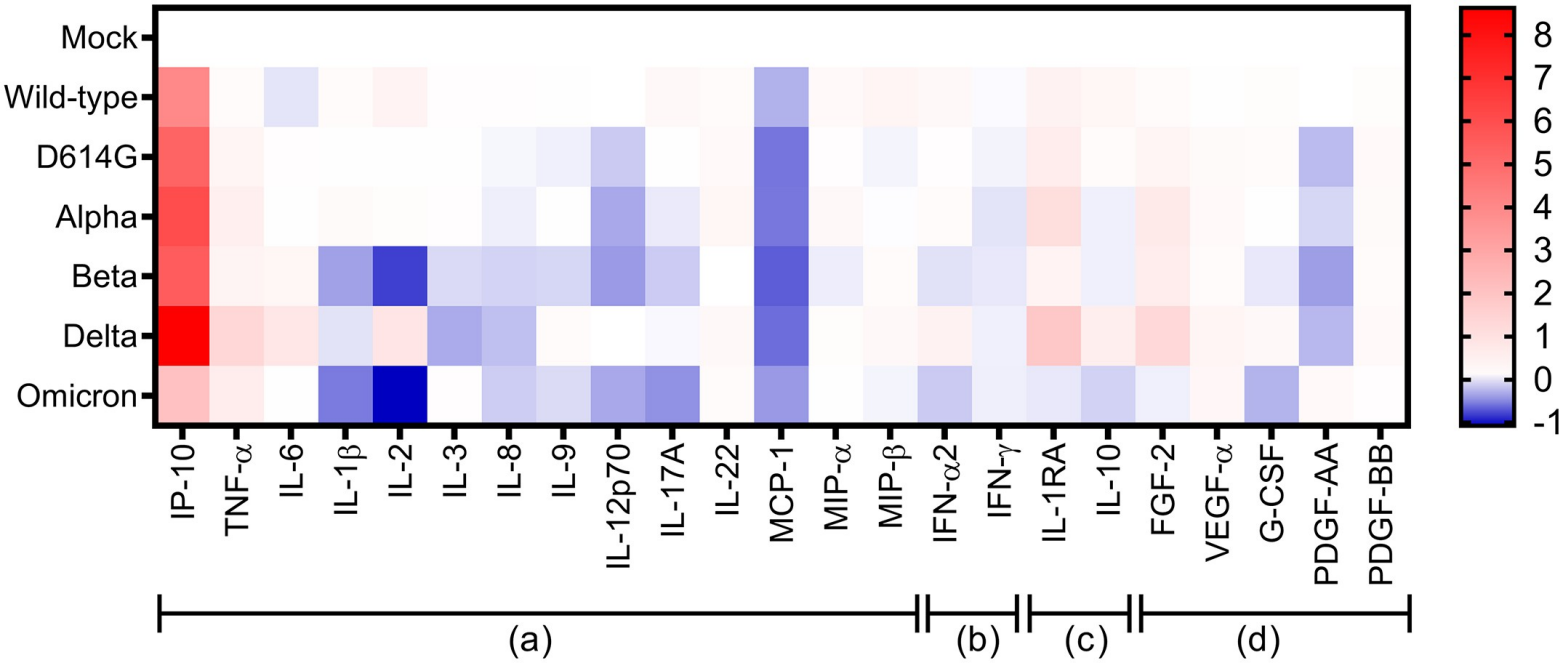
Heatmap of cytokine expression in Calu-3 cells infected with different SARS-CoV-2 VOCs at 48 hpi. The cytokine concentrations were relatively transformed into log2 fold change value and scale in comparison to mock infection. Four main cytokine categories are presented, including pro-inflammatory cytokines/chemokines **(a)**, interferons **(b)**, anti-inflammatory cytokines **(c)**, and growth factors **(d)**.

The levels of pro- and anti-inflammatory cytokines/chemokines, along with growth factors induced by each SARS-CoV-2 variant, are depicted in a log_2_ heat map (Fig 7). Notably, infection with all SARS-CoV-2 variants led to a significant induction of IP-10/CXCL10 in Calu-3 cells, with the highest expression observed following infection with the Delta variant. Additionally, the stimulation of other inflammatory mediators, such as TNF-α, IL-6, and IL-1RA as well as a growth factor, FGF-2, were emphasized. Of note, infection of Calu-3 cells with Omicron led to limited induction of IP-10/CXCL-10.

## Discussion

Assessing the potential risk of severe illness or mortality associated with SARS-CoV-2 variants requires a thorough investigation of their phenotypic characteristics. In this study, we conducted head-to-head infections of Calu-3 human bronchial epithelial cells with a range of variants, including the wild-type, D614G, Alpha, Beta, Delta, and Omicron, to gain valuable insights into these characteristics. Notably, at 24 hpi all variants except Omicron had significantly higher proportions of NP-positive cells compared to wild-type. This suggests that D614G, Alpha, Beta, and Delta all have enhanced viral replication capabilities and a greater ability to establish infection in the early stages compared to the original strain. All variants established peak viral entry by 48 hpi, except for the Omicron, which continued to enter cells up to 72 hpi, indicating a slightly prolonged entry process (Fig 1a and 1b). This observation is consistent with previous research demonstrating that a reduction in the S1 and S2 cleavage is a major contributing factor to the inefficient virus entry observed with the Omicron variant, particularly in cell types with higher expression of TMPRSS2 [21]. The Omicron spike has been shown to exhibit inadequate utilization of the cellular protease TMPRSS2, leading to reduced fusogenisity and a tendency to exploit the slower, acid-dependent endocytic pathway for cell entry [48]. A study in 2020 revealed that children had notably lower levels of ACE2 and TMPRSS2 expression in both the upper and lower airways compared to adults [49]. This observation coupled with the Omicron variants’s reduced reliance on the TMPRSS2-dependent entry pathway may provide an explanation for the increased frequency of children hospitalization observed during the Omicron wave compared to previous waves [50]. After reaching the peak at 48 hours, the proportion of NP-positive cells declined in the Calu-3 cells infected with D614G, Alpha, Beta, and Delta variants at 72 hours post-infection, with Delta-infected cells reduced the highest percentage by almost 30% (Fig 1a and 1b), suggesting a possibility of decreased cell susceptibility to SARS-CoV-2 infection, particularly with the D614G, Alpha, Beta, and Delta variants at 72 hours post-infection. Lucía Gutiérrez-Chamorro et al (2021) reported a decreased level of ACE2 mRNA in nasopharyngeal swabs of COVID-19 patients on day 3 compared to day 1 [51]. Another study conducted in Vero E6 and hACE2-HeLa cells also confirmed that the downregulation of ACE2 expression in SARS-CoV-2 infection was regulated by the presence of spike protein [52]. Our findings support the hypothesis that mutation profile of spike protein among variants might affect to cell susceptibility in later time point of infection by decreasing level of ACE2 receptor expression. Further study to investigate the impact of different mutation profiles in variant’s spike in receptor regulation of infected cells would be interesting.

In terms of virus replication kinetics, in our model the results showed that the Delta variant released the highest number of infectious virus particles at 12 hours of infection. This, along with the greater percentage of NP-positive cells, indicates that the Delta has a clear advantage over variants in terms of virus entry and early progeny virus generation. However, the lower amount of virus particles at 24 hours of the Delta variant compared to the D614G and Alpha variant, as well as the wild-type at 48 hours, is likely due to the pronounced syncytia formation in Delta variant-infected cells (Fig 3). This distinctive morphological feature in Calu-3 cells may trigger virus transmission through cell-to-cell pathways. Our findings are supported by a previous co-culture cell study which demonstrated that SARS-CoV-2 infection occurs more prevalently through cell-to-cell spread than in cell-free mechanisms [30]. Furthermore, all variants, except for Omicron, replicated effectively and reached the peak of infectious titers at 48 hours post-infection. These infection kinetic results indicate that the common D614G mutation within the spike gene of those main variants facilitates faster virus transmission [53]. Additionally, the characteristic P681R mutation in the Delta variant could possibly responsible for enhancing the virus entry through rapid cleavage of spike protein into S1 and S2 subunits compared to wild-type and Alpha variant [54]. In contrast, we observed that the Omicron variant released the lowest number of infectious particles at 12, 24, and 48 hpi indicating a slower replication rate compared to all other variants in Calu-3 cells. These findings align with several studies that have also reported the less effective growth kinetic of the Omicron than the wild-type and Delta variants in TMPRSS2-expressed cells [55,56]. However, by 72 hpi, the replication of Omicron reached levels comparable to other variants. These findings suggest a distinct temporal pattern of viral replication for the Omicron variant. The delayed viral replication observed in the early stages of infection suggests a potential altered, and slightly hampered, interaction between the Omicron variant and host bronchial epithelial cells, leading to a slower, but still effective, viral spread within the respiratory system. The subsequent catch-up in viral replication observed at 72 hpi suggests that the Omicron variant may have developed compensatory mechanisms to overcome these early entry or replication barriers. Additionally, the interplay between this viral infection and a delayed innate immune system might be also a mechanism that contributes to different levels of suppression to its replication in infected cells and antiviral state in the local environment, as outlined in earlier studies [40,57]. Moreover, the ability to ultimately achieve similar replication levels as other variants highlights the adaptability and resilience of the Omicron variant.

To support the associated pathological effects, we observed the prominent formation of multinucleated giant cells or syncytia in Calu-3 cells following infection with the Delta variant, in comparison to other variants, including Omicron. Syncytia formation is a process characterized by the fusion of infected cells expressing the viral S protein with neighboring cells expressing ACE2, facilitating direct cell-to-cell transfer of viral components. This fusion process is known to be accelerated by the TMPRSS2 protease [28]. Syncytia formation allows viruses to disseminate faster and more efficiently compared to the conventional mode of viral spread, where viral components are first released into the extracellular space before infecting target cells. The rapid and efficient cell-to-cell transmission via syncytia formation confer an advantage to the virus, allowing it to evade the host’s humoral immune system, making it less accessible to neutralizing antibodies [58,59]. Several studies have shown that syncytia can target lymphocytes by mediating homotypic or heterotypic cell-in-cell mediated death (death of internalized cells in acidified vacuoles), thereby potentially contributing to lymphopenia in COVID-19 patients [60–62]. Post-mortem lung tissues obtained from COVID-19 patients who died also revealed the formation of syncytia in fused pneumocytes, indicating productive infection. These studies confirmed the presence of large multinucleated pneumocytes as a significant feature of severe lung damage [24,63]. Syncytia with more nuclei or those that are larger in size are more likely to internalize lymphocytes [60]. Notably, our investigation revealed distinct patterns of syncytia formation among SARS-CoV-2 variants. Specifically, the Alpha and Delta variants showed significantly higher levels of small syncytia formation, while the D614G and Alpha variants exhibited significantly elevated levels of large syncytia compared to the wild-type. The Delta variant demonstrated an upregulation of large syncytia formation as well, but the difference was not statistically significant. In contrast, the Omicron variant exhibited significantly lower numbers of small syncytia, and no detectable large syncytia, compared to the wild-type. The Omicron variant’s attenuated syncytia formation may help explain its lowered pathogenicity compared to the Delta variant. Previous studies have also described the weakening of fusion activity upon Omicron variant infection in TMPRSS2-expressed cells that correlated to impaired syncytia formation [21,56,64]. Additionally, the alteration within spike protein is likely the main determinant factor in the defect of Omicron to form syncytia, which has been indicated in a cell-cell fusion experiment [48]. Furthermore, at 48 hours, the obvious CPE of SARS-CoV-2-infected Calu-3 cells, indicated by present of rounding and detached cells, was observed in the Delta variant, followed by the Alpha, Beta and D614G. However, the wild-type and Omicron variant infection yielded very less CPE (Fig 3). This result was supported by the previous study that demonstrated Calu-3 cells detachment at 4–5 days following infection with Omicron variant at MOI of 0.1 [47]. Another in-vitro experiment of SARS-CoV-2 variants at lower infectivity titer (MOI: 0.0001) in Calu-3 cells also showed the Delta variant triggers CPE at the earliest time, followed by Gamma, Alpha, non-VOC (B.1.1) and Beta variants, however, the CPE rate in the Omicron variant is remarkable weak [65]. Our findings revealed the various number of syncytia cells among SARS-CoV-2 variants potentially trigger the different CPE level in infected cells that might reflect the grade of cell death. However, a limitation of this study is the lack of confirmation data for cell death and cell-type involvement, necessitating further investigation into the cell death profiles among SARS-CoV-2 variants.

Severe COVID-19 is linked to lung epithelial cell destruction, which can stem from both viral cytopathic effects and immune-mediated pathology. Syncytia formation has been linked to the activation of cyclic GMP–AMP synthase-stimulator of interferon genes (cGAS-STING) pathway and the nuclear factor-kappaB (NF- κB) transcription factor [66,67], which in turn regulates the induction of various pro-inflammatory cytokines [68,69]. Furthermore, syncytia-mediated cell death via apoptosis or pyroptosis can also promote the production of proinflammatory cytokines [58,70]. While numerous studies have reported the serum levels of various cytokines/chemokines in COVID-19 patients, both in mild and critical stages, limited correlation has been observed with those in bronchoalveolar lavage fluid (BALF) [71]. Furthermore, the distinct induction of inflammatory mediators in pulmonary epithelial cells following infection with different SARS-CoV-2 variants remains unclear. We characterized the profiles of cytokines produced by Calu-3 cells subsequent to infection with the wild-type, D614G, Alpha, Beta, Delta and Omicron variants. Our findings revealed that the Delta variant significantly upregulated the highest levels of pro-inflammatory cytokines in Calu-3 cells. Notably, the levels of IP-10, TNF-α, and IL-6 were significantly elevated compared to the wild-type. IP-10, known for attracting effector T cells to inflamed airway epithelia [72], has been associated with cytokine storm syndrome and systemic inflammation in severe COVID-19 cases [35,73,74]. Elevated IL-6 levels contribute to severe lung damage, and both IL-6 and TNF-α play crucial roles in cytokine storm, ARDS, and mortality in COVID-19 patients [75–77]. Our study indicates that the Delta variant has the ability to induce ARDS-related cytokines in Calu-3 lung epithelial cells, while the wild-type, D614G, Alpha, Beta, and Omicron variants showed comparable levels of these cytokines.

In addition to pro-inflammatory cytokines/chemokines, infection with the Delta variant also significantly stimulated the production of an anti-inflammatory mediator, IL-1RA. Elevated levels of inhibitory cytokines, such as IL-1RA and IL-10 has been observed in COVID-19 patients with severe outcomes, highlighting their association with disease progression [78,79]. These anti-inflammatory cytokines act as negative feedback regulators, aiming to limit the hyperactive immune response triggered by viral infection. Accordingly, the robust induction of IL-1RA detected during Delta variant infection indicates the activation of regulatory network that may help minimize immunopathology and limit cellular damage. Furthermore, the synthesis of IL-10 manifests potent immunosuppressive effects on monocytes, macrophages (cells exhibiting heightened IL-10R expression), and dendritic cells [80].

In addition to pro- and anti-inflammatory cytokines, Delta variant infection increased levels of growth factors FGF-2 and VEGF-A. The FGF-2 promotes angiogenesis and inflammation, and it also attracts immune cells to angiogenesis sites [81,82]. This aligns with a study showing increased lung blood vessel growth in fatal COVID-19 cases correlated with upregulated FGF2 gene expression [83]. Notably, FGF-2 has been implicated in the pathogenesis of various viruses, including Kaposi’s sarcoma-associated human herpes virus 8 (HHV8), cytomegalovirus (CMV), hepatitis C virus (HCV), human papilloma virus (HPV), human T-cell lymphotropic virus type 1 (HTLV-1), and Middle East respiratory syndrome coronavirus (MERS-CoV) [83,84]. Furthermore, our model found elevated VEGF-A upon Delta variant infection, which is known to be induced by hypoxia, a common symptom of severe COVID-19 [85,86]. Indeed, the role of VEGF extends beyond angiogenesis and vascular permeability and includes an important contribution to the inflammatory process. A clinical investigation by Ackermann et al. in 2020 has revealed the upregulation of angiogenesis-related genes, including VEGFA, in the lung tissues of COVID-19 patients who experienced fatal outcomes [87]. This upregulation of VEGF-A has been proposed to induce plasma extravasation, leading to pulmonary edema, which is a characteristic feature of ARDS in COVID-19 patients [88]. In line with these findings, our model’s observation of elevated FGF-2 and VEGF-A following Delta variant infection suggests their potential involvement in exacerbating respiratory distress symptoms associated with COVID-19. This information emphasizes the detrimental impact that high levels of FGF-2 and VEGF-A could have on the respiratory function of COVID-19 patients.

Although our study focuses solely on Calu-3 cells and lacks the complete interplay of inflammatory responses in the other lung cell types, such as endothelial cells, we were still able to identify some critical inflammatory markers. These markers are potential indicators to distinguish impacts among different SARS-CoV-2 VOCs, demonstrating the effectiveness of our approach in understanding the virus’s pathogenicity.

Our model is in agreement to the real world observations [89,90] that the Omicron variant exhibits attenuated pathogenicity compared to other variants, such as the wild-type, D614G, Alpha, Beta, and Delta. Many studies have demonstrated the weakened fusogenicity during Omicron infection in cell-based experiments that might be correlated to less disease severity [21,55,56]. This decreased capability can be attributed to the mutation profile of the Omicron variant. Among the 37 amino acid mutations within the spike protein compared to Wuhan-Hu-1 strain, several important altered amino acids have been identified. These mutations affect interprotomer electrostatic contacts between S1 and S2 subunit (N764K, T547K, and N856K) and help to stabilize the S1 and S2 prefusion conformation (N679K/P681H) [91]. Similar correlations between viral fusogenicity and pathogenicity have been described in studies on HIV and measles virus [92]. Therefore, in our model the weaker ability of the Omicron variant to trigger cell-cell fusion or syncytia formation, along with crucial factors including diminished growth kinetics, and reduced release of inflammatory cytokines could contribute to its lower viral pathogenicity. In contrast, our study highlights the severe damage caused by the Delta variant in Calu-3 lung epithelial cells. These findings align with clinical studies showing increased mortality risk with Alpha, Beta, and Gamma variants, and especially the Delta variant, compared to the wild-type strain [7]. On the other hand, patients infected with the Omicron variant tend to experience milder symptoms, fewer hospitalizations, and lower mortality rates [89,90,93]. However, other heterogeneous host factors such as age, sex, vaccine status, and geographical parameters could also interfere the clinical disease severity of COVID-19 [6]. All in all, comparative *in vitro* studies on SARS-CoV-2 variant infection kinetics, syncytia formation, and inflammatory biomarkers in specific cell types can serve as valuable predictive indicators for variant pathogenicity, with potential application to future emerging viruses.

The primary limitation in our study is its reliance on the Calu-3 cell model for investigating SARS-CoV-2 variants, particularly Omicron, with a specific focus on the pathogenicity in the human lower respiratory tract. However, this method may not fully encompass the virus’s distinctive characteristics in the upper respiratory system, including the nasal epithelium, as indicated by other researches [48,94–96]. Additionally, our examination of the Omicron BA.2 subvariant, potentially overlooking variations in pathogenicity compared to other subvariants like BA.1 and BA.5 [97,98]. To thoroughly assess the virus’s pathogenicity, future studies should explore a variety of cell models and include biomarkers such as IFN-1A, IFN-β, IFN-λ, and CCL-5 [43,99,100]. Employing this approach may help establish whether a single cell line adequately represent the virus’s behavior.

## Conclusions

Based on the findings of our study, the different SARS-CoV-2 variants exhibit distinct characteristics in Calu-3, human lung epithelial cells model. The B.1.36.16 (D614G), Alpha, Beta, and Delta variants demonstrated enhanced virus entry and higher infection rates compared to the wild-type strain. These variants have also displayed a similar pattern of virus replication which is 48 hours as the peak virion production. Notably, the Delta variant has triggered the most significant formation of syncytia, followed by the Alpha variant. Opposed to these results, in our model the Omicron variant exhibited a lower infectivity rate, slower replication kinetic, and fewer syncytia formations than other VOCs and wild-type strains. In terms of immunopathology response, the Delta variant elicited a significant upregulation of specific profiles of inflammatory cytokines (IP-10, TNF-α, IL-6, and IL-1RA) and growth factors (FGF-2 and VEGF-A), indicating its potential for high pathogenicity in lung epithelial cells. Conversely, these inflammatory mediators are less secreted in the Omicron variant infection. Together, our results are in line with cytopathology observations in lung biopsies of COVID-19 patients with severe pneumonia, suggesting that comparative in vitro studies of SARS-CoV-2 variant infection kinetics, syncytia formation, and inflammatory biomarkers may serve as valuable predictive indicators for variant pathogenicity.

## Supporting information

S1 Table The concentrations of each immune mediator released in Calu-3 cell supernatants upon infection with SARS-CoV-2 variants 48 hours after infection. Values indicated the average from triplicates experiment (pg/mL).
(DOCX)
