# Supplementary material for: Infection kinetics, syncytia formation, and inflammatory biomarkers as predictive indicators for the pathogenicity of SARS-CoV-2 Variants of Concern in Calu-3 cells
Source: PLoS One. 2024 Apr 3;19(4):e0301330. doi: 10.1371/journal.pone.0301330 (PMC10990222; doi:10.1371/journal.pone.0301330)
Supplement: S1 Table — Values indicated the average from triplicates experiment (pg/mL). (DOCX) [file pone.0301330.s001.docx]

**S1 Table.** **The concentrations of each immune mediator released in Calu-3 cell supernatants upon infection with SARS-CoV-2 variants 48 hours after infection. Values indicated the average from triplicates experiment (pg/mL).**

| **No** | **Analyte** | **Mean** | **Mock** | **Wild-type** | **D614G** | **Alpha** | **Beta** | **Delta** | **Omicron** |
| --- | --- | --- | --- | --- | --- | --- | --- | --- | --- |
| 1 | IP-10 | Geometric mean | 8.23 | 129.30 | 306.00 | 538.70 | 362.70 | 3236.00 | 34.56 |
|  |  | SD | 1.08 | 1.38 | 1.17 | 1.35 | 1.22 | 1.26 | 1.10 |
| 2 | TNF-α | Geometric mean | 10.29 | 11.17 | 12.84 | 14.73 | 13.20 | 25.73 | 15.93 |
|  |  | SD | 1.05 | 1.12 | 1.05 | 1.05 | 1.07 | 1.11 | 1.10 |
| 3 | IL-6 | Geometric mean | 661.60 | 747.70 | 833.20 | 754.90 | 983.50 | 1391.00 | 778.20 |
|  |  | SD | 1.18 | 1.07 | 1.10 | 1.05 | 1.10 | 1.09 | 1.07 |
| 4 | IL-1β | Geometric mean | 1.96 | 2.11 | 1.97 | 2.17 | 1.49 | 1.81 | 1.33 |
|  |  | SD | 1.13 | 1.10 | 1.06 | 1.12 | 1.15 | 1.06 | 1.26 |
| 5 | IL-2 | Geometric mean | 0.25 | 0.34 | 0.26 | 0.27 | 0.15 | 0.46 | 0.12 |
|  |  | SD | 1.17 | 1.52 | 1.41 | 1.43 | 1.19 | 1.30 | 1.30 |
| 6 | IL-3 | Geometric mean | 1.71 | 1.78 | 1.71 | 1.79 | 1.53 | 1.34 | 1.76 |
|  |  | SD | 1.14 | 1.30 | 1.30 | 1.11 | 1.19 | 1.18 | 1.13 |
| 7 | IL-8 | Geometric mean | 9359.00 | 9731.00 | 9110.00 | 8907.00 | 8218.00 | 7755.00 | 8075.00 |
|  |  | SD | 1.10 | 1.13 | 1.03 | 1.09 | 1.09 | 1.27 | 1.16 |
| 8 | IL-9 | Geometric mean | 17.08 | 17.45 | 16.31 | 17.13 | 15.11 | 18.34 | 15.34 |
|  |  | SD | 1.06 | 1.06 | 1.09 | 1.08 | 1.07 | 1.05 | 1.06 |
| 9 | IL-12p70 | Geometric mean | 1.51 | 1.51 | 1.29 | 1.16 | 1.12 | 1.51 | 1.16 |
|  |  | SD | 1.16 | 1.16 | 1.16 | 1.14 | 1.08 | 1.16 | 1.14 |
| 10 | IL-17A | Geometric mean | 2.94 | 3.44 | 2.99 | 2.76 | 2.52 | 2.88 | 2.13 |
|  |  | SD | 1.04 | 1.11 | 1.11 | 1.40 | 1.28 | 1.24 | 1.38 |
| 11 | IL-22 | Geometric mean | 19.98 | 21.56 | 22.70 | 24.52 | 19.96 | 23.49 | 21.74 |
|  |  | SD | 1.02 | 1.23 | 1.06 | 1.06 | 1.10 | 1.18 | 1.08 |
| 12 | MCP-1 | Geometric mean | 34.46 | 27.41 | 22.94 | 23.10 | 21.37 | 22.40 | 25.50 |
|  |  | SD | 1.26 | 1.39 | 1.49 | 1.34 | 1.21 | 1.44 | 1.13 |
| 13 | MIP-1α | Geometric mean | 13.55 | 15.41 | 14.16 | 15.84 | 12.85 | 14.51 | 13.75 |
|  |  | SD | 1.03 | 1.11 | 1.03 | 1.03 | 1.03 | 1.05 | 1.04 |
| 14 | MIP-1β | Geometric mean | 1.70 | 2.12 | 1.65 | 1.69 | 1.85 | 1.97 | 1.64 |
|  |  | SD | 1.12 | 1.10 | 1.06 | 1.10 | 1.25 | 1.07 | 1.06 |
| 15 | IFN-α2 | Geometric mean | 14.00 | 16.15 | 14.49 | 15.34 | 12.81 | 18.74 | 11.97 |
|  |  | SD | 1.07 | 1.17 | 1.28 | 1.01 | 1.24 | 1.13 | 1.08 |
| 16 | IFN-γ | Geometric mean | 15.80 | 15.62 | 15.22 | 14.58 | 14.73 | 15.08 | 15.05 |
|  |  | SD | 1.03 | 1.02 | 1.02 | 1.06 | 1.01 | 1.07 | 1.03 |
| 17 | IL-1RA | Geometric mean | 8.40 | 11.35 | 13.07 | 17.99 | 11.10 | 30.21 | 7.85 |
|  |  | SD | 1.13 | 1.17 | 1.05 | 1.14 | 1.15 | 1.04 | 1.06 |
| 18 | IL-10 | Geometric mean | 1.21 | 1.46 | 1.30 | 1.15 | 1.15 | 1.77 | 1.06 |
|  |  | SD | 1.07 | 1.11 | 1.12 | 1.14 | 1.14 | 1.09 | 1.08 |
| 19 | FGF-2 | Geometric mean | 290.50 | 313.80 | 360.40 | 468.80 | 440.40 | 691.40 | 277.80 |
|  |  | SD | 1.15 | 1.10 | 1.07 | 1.03 | 1.14 | 1.08 | 1.04 |
| 20 | VEGF-A | Geometric mean | 832.30 | 839.60 | 918.90 | 955.80 | 908.80 | 1044.00 | 1002.00 |
|  |  | SD | 1.01 | 1.01 | 1.04 | 1.06 | 1.03 | 1.05 | 1.04 |
| 21 | G-CSF | Geometric mean | 21.28 | 22.69 | 23.09 | 21.44 | 19.84 | 24.85 | 17.03 |
|  |  | SD | 1.10 | 1.19 | 1.06 | 1.07 | 1.14 | 1.02 | 1.21 |
| 22 | PDGF-AA | Geometric mean | 52.23 | 52.15 | 42.71 | 46.29 | 39.22 | 42.34 | 59.54 |
|  |  | SD | 1.03 | 1.05 | 1.01 | 1.10 | 1.05 | 1.07 | 1.05 |
| 23 | PDGF-BB | Geometric mean | 541.20 | 574.10 | 618.30 | 608.10 | 585.00 | 613.40 | 557.80 |
|  |  | SD | 1.07 | 1.03 | 1.03 | 1.06 | 1.02 | 1.03 | 1.08 |
